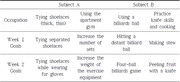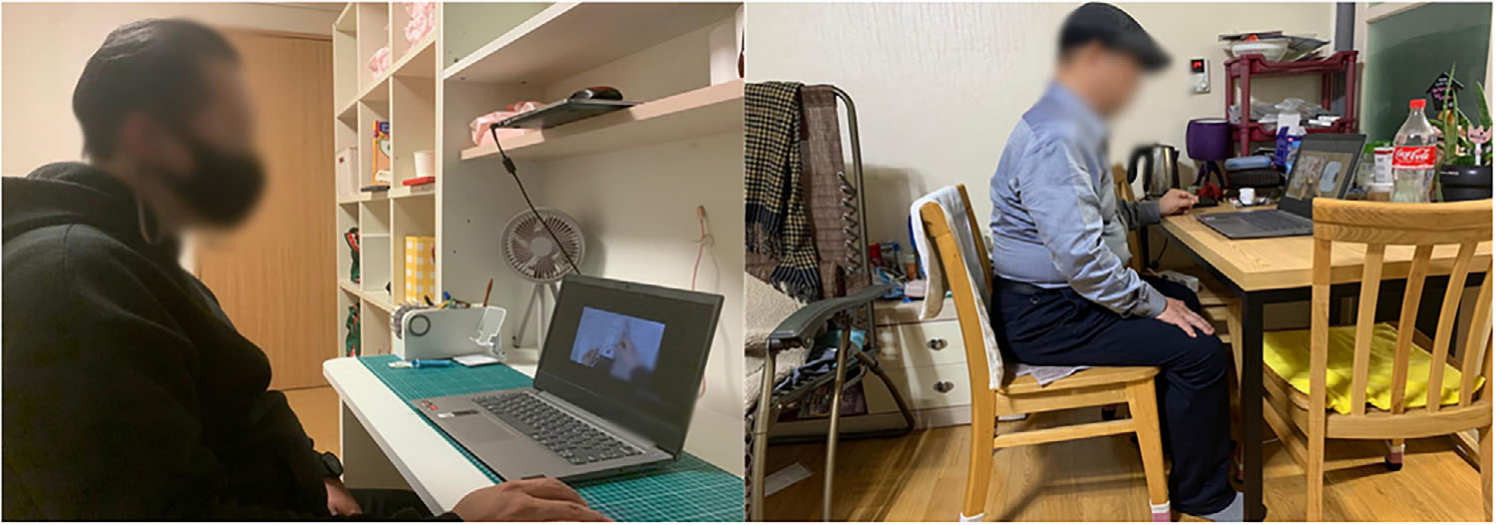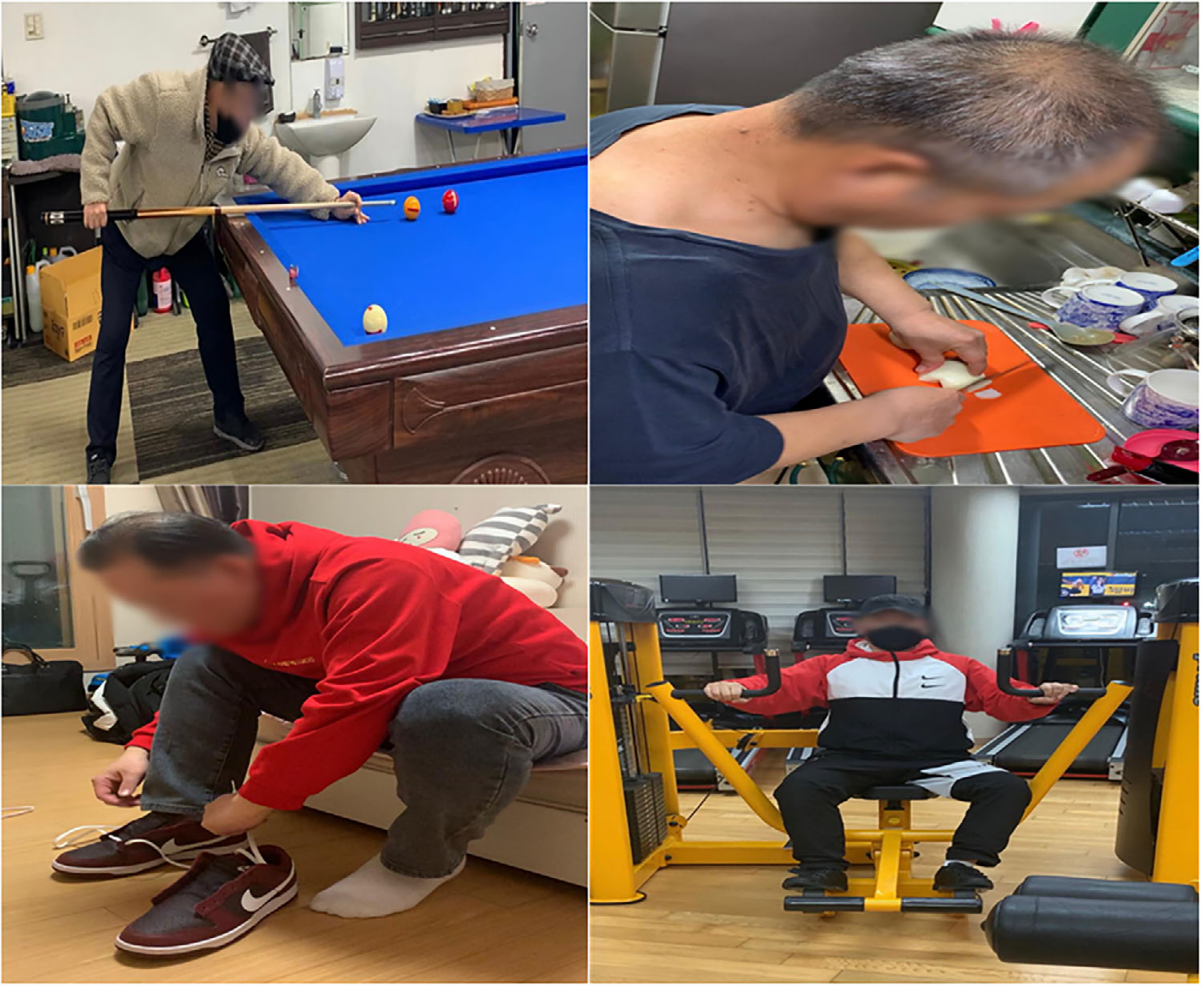# The Effects of Action Observation Training Combined With Occupation‐Based Intervention through Home Visits on Occupational Performance and Hand Function in Community‐Dwelling Patients with Stroke

**DOI:** 10.1002/alz.094650

**Published:** 2025-01-09

**Authors:** Jong‐Hyeon Kim, Jin‐Hyuck Park

**Affiliations:** ^1^ Yonsei University, Gangwon state, Wonju Korea, Republic of (South); ^2^ Soonchunhyang University, Chungcheongnam‐do Province, Asan Korea, Republic of (South)

## Abstract

**Background:**

Since January 2023, South Korea has launched a pilot program of home‐based rehabilitation services for patients with acute and chronic conditions. However, research on the effectiveness of occupational therapy for homebound stroke patients, aimed at promoting independence and activity participation, is limited. Additionally, previous action observation training studies in home settings often overlooked clients' occupational priorities and meanings. Therefore, this study investigates the effect of combining action observation training with occupational therapy through home visits on occupational performance and hand function in community‐dwelling stroke patients.

**Method:**

In this study, which employed a mixed methods research design, the quantitative component utilized a single‐subject ABA' design, complemented by qualitative narrative interviews. The intervention involved two stroke outpatients, who participated in ten sessions conducted five times a week over two weeks. Each session began with the participants watching edited videos on the researcher’s laptop for 10 minutes in a quiet room, followed by 50 minutes of self‐training using selected equipment and tools, while imitating the actions from the videos. Assessment tools included the Purdue Pegboard Test and Pinch Gauge for evaluating hand function, while occupational performance was measured using the COPM pre‐ and post‐intervention.

**Result:**

First, twol participants showed clinically significant improvements with a score increase of two or more points in occupational performance and satisfaction. Second, twol participants demonstrated an improvement in the assembly task of the Purdue pegboard test during the intervention phase compared to the baseline phase, but this improvement was not maintained during the follow‐up baseline phase. Third, in the Pinch gauge, two participants showed an improvement in the affected side from the baseline to the intervention phase, and this improvement was maintained until the follow‐up baseline phase. However, there were no significant changes observed in the unaffected side. Fourth, qualitative research findings revealed that the participants exhibited an improvement in occupational performance, occupational motivation, occupatioanl patterns, and self‐efficacy.

**Conclusion:**

Action observation training combined with home‐based interventions can be easily implemented at home without significant effort from patients post‐discharge. Therefore, it offers an alternative method for occupational therapy services, serving as a preparatory step before engaging in occupational activities.